# Satisfaction with Physical Activity among Students in the Last Cycle of Primary Education in Extremadura

**DOI:** 10.3390/ijerph19116702

**Published:** 2022-05-31

**Authors:** Jorge Rojo-Ramos, María José González-Becerra, Santiago Gómez-Paniagua, José Carmelo Adsuar

**Affiliations:** 1 Health, Economy, Motricity and Education (HEME) Research Group, University of Extremadura, Avda. de la Universidad s/n, 10003 Cáceres, Spain; jadssal@unex.es; 2 BioẼrgon Research Group, University of Extremadura, 10003 Cáceres, Spain; mgonzalexd@alumnos.unex.es (M.J.G.-B.); sgomezpa@alumnos.unex.es (S.G.-P.)

**Keywords:** physical activity, satisfaction and enjoyment

## Abstract

Satisfaction with physical activity during Physical Education classes leads to improved health and adherence to future healthy lifestyle habits, in addition, higher levels of physical activity have also been widely associated with higher academic achievement in primary school. To find out how satisfied Extremadura students are with the physical activity they perform, a questionnaire was distributed with different items related to positive and negative feelings they may feel during the practice. The differences between the items of the questionnaire and the total median scores according to sex and center location were analyzed and found to be present in all the items that refer to positive feelings. Additionally, the relationship between age and the mean score obtained through the questionnaire was evaluated, without discovering any significant correlations. The results showed that students are generally satisfied with the physical activity they practice in their classes and that this has benefited their academic performance.

## 1. Introduction

Today, the population attaches great importance to healthy lifestyle habits [1,2], but acquiring them is not an easy task [3]. Established organizations such as the World Health Organization make different recommendations for different types of the population [4]. In the case of children, they recommend 60 min of moderate or vigorous aerobic activity per day. Physical Education has become a tool to encourage and promote this type of habit among young people and has the ability to raise awareness of the benefits of physical activity (PA) at different levels. This tool can be very useful if we consider that children and young people spend most of their time in educational environments which, for the most part, also form their social environment; this is where they form their friendships and create the opportunity to engage in PA and adhere to it, in order to continue the practice outside the educational environment [5]. Nevertheless, it is not easy to get young people to adhere to the physical activity recommended by the WHO [6]. Schools should represent a means to promote messages such as avoiding inactivity or sedentary lifestyles and that tell children that these health problems are linked to increases in adiposity, poorer cardio-metabolic health and physical fitness, or shorter sleep duration [7].

Physical Education is a central subject in the education and integral formation of children throughout the school year, providing opportunities for children to acquire the physical, cognitive, and emotional skills necessary for their later success [8]. Children learn, perform, and develop new movement patterns in these programs, as well as a better understanding, respect, and appreciation of themselves and those around them [9]. After physical activity, the three most pronounced changes were in self-esteem, mood, and motor skills [10]. In his research, Kipp states that adolescents’ life satisfaction is determined by high self-esteem, which is largely influenced by physical activity [11].

Physical Education classes have been increasingly important in the educational setting throughout time, with PE and student pleasure in the class recognized as crucial to students’ learning passion [12]. The level of fun is a major aspect in motivating students to participate in school PE, and the higher the level of enjoyment, the more committed the students are to the topic [13]. Motivation has a direct impact on the development of healthy lifestyle habits and produces adhesion to the practice of Physical Activity in the non-academic context [14], an effect that PE teachers should consider when providing positive experiences that impact extracurricular sports practice, maintenance, and adherence in adult life [15]. Having the essential skills and information is not enough in the academic arena to achieve goals. In their study, Fraile-García et al. found that students’ grades in PE were higher because enjoyment was associated with physical activity, motor self-efficacy, and the amount of physical activity performed, with satisfaction being the variable with the most weight and most associated with student performance in PE [16]. Motivation, according to Järvenoja and Järvelä, is a person’s process of engaging and sustaining himself in a task that will lead him to achieve his goals [17].

Motivation is vital in learning, motor skill acquisition, and performance in both sports and PA, and it adds to the value of self-effort and tenacity as factors that may influence personal fulfillment [14]. There are two sorts of motivation: extrinsic motivation (working for the purpose of earning benefits from outside sources) and intrinsic motivation (working for the purpose of receiving rewards from within), which entails performing things just for pleasure, enjoyment, interest, or fulfillment [18]. As a result, when a student is happy and having fun in class, he or she is more likely to be intrinsically motivated, which means more involvement in PE and even more physical activity practice in free time [19]. When there is a task-oriented atmosphere, there is a good impact on children’s cognition, satisfaction, and practice of extracurricular PA, according to Gråstén and Watt [20]. Intrinsic motivation determines the degree of satisfaction in Physical Education, according to Navarro-Patón et al. In this sense, if a student develops intrinsic motivation for PA, it is very likely that he or she will develop a high level of adherence to the practice of physical sports activities in the future [21] because the effects of a climate of high involvement in PE class were shown to increase success, effort, and satisfaction of PA adoption in students [22,23].

Motivation may have ramifications outside of sports. Some are good, such as striving or enduring, while others are negative, such as boredom, which is linked to external motivation [24]. This should give the PE teacher the opportunity to take timely and important actions in the teaching-learning process, which can be done through the use of diverse strategies, positive feedback, or task diversity [18]. Teachers that employ instructional methods and didactic tactics that encourage students to engage and to be propositional and involved in task design and decision making obtain higher levels of involvement and enjoyment in physical exercise [25]. Another method might be to consider group sports as a kind of physical activity, as Zullig and White discovered in their study that involvement in these activities enhances feelings of contentment [26]. Other negative repercussions can impact public health and educational issues, such as bullying [27].

Satisfaction can be understood as a person’s personal cognitive assessment of life and its domains, considering the quality of his or her life, expectations and aspirations, and the goals achieved, based on the person’s own criteria and which is realized in a favorable way [28]. Life satisfaction can be considered a reliable indicator of personal well-being and psychological development, with self-satisfaction, family satisfaction, and school satisfaction as fundamental determinants [29,30,31].

The purpose of this study was to determine how satisfied kids in the last cycle of elementary school were with the physical exercise that they carry out in Physical Education classes. The Physical Activity Children Enjoyment Scale (PACES) questionnaire, which has been translated into Spanish [32], was utilized to gather data for this study. It consists of 16 items that start with the sentence “when I am active (performing physical activity, physical exercise, sport …)” and assesses positive or negative sentiments of acceptance or rejection toward physical activity. The last cycle of primary school was chosen because it is seen as a critical age amid compulsory education for determining whether kids are satisfied or not while engaging in physical activity and, as a result, for changing techniques in bad circumstances.

## 2. Materials and Methods

### 2.1. Participants

A total of 545 Physical Education pupils in the final cycle of elementary school were included in the study. Boys made up 50.8 percent of the total, while females made up 49.2 percent. In terms of geography, 55.6 percent of the centers were in rural areas while 44.4 percent were in urban areas; defining the terms “urban” or “rural” according to whether the population exceeds 10,000 inhabitants [33]. The sample was judged and balanced in terms of sex and center location, the two key factors under investigation.

The variables gender, center location, province, or school year are shown in Table 1 below. The age distribution of the participants is also shown in Table 1.

### 2.2. Instruments and Measures

A Google Forms form was the tool used to obtain the sociodemographic data of the participants. To measure the level of satisfaction with physical activity, we used the Spanish version of the Physical Activity Children Enjoyment Scale (PACES) questionnaire [32], and adapted it to the young population. This instrument is composed of 16 items preceded by the phrase “when I am active (doing physical activity, physical exercise, sport...)”. It uses a Likert-type scale with values ranging from 1 to 5, with 1 denoting “complete disagreement” and 5 denoting “complete agreement.” Of the 16 items, 9 refer to positive feelings of acceptance of physical activity and 7 refer to negative feelings or rejection of physical activity. The negative items were inverted since the application of the scale yields a score from the sum of all the items, with 16 being the minimum value corresponding to a low level of enjoyment of the physical activity and 80 being the value equivalent to the maximum enjoyment of the physical-sports activity.

### 2.3. Procedures

The Google Forms tool was used to develop the questionnaire, incorporating the sociodemographic questions and the PACES. It was decided to use an e-questionnaire because it facilitated delivery, saved time, and all the responses were stored in the same database, obtaining a higher return rate [34].

First, the Department of Education and Employment of the Regional Government of Extremadura’s directory of educational centers (https://ciudadano.gobex.es/ciudadanoportlet/printpdf/pdf?typepdf=3443&idDirectorio=775, accessed on 25 February 2021) was accessed and all the public educational centers that taught primary education were selected. Following that, an e-mail was issued to all of the school directors telling them of the study’s purpose and included an informed parental permission form, so that if the school agreed to collaborate, the students would have to have signed parental consent to participate in the study. At the end of the e-mail, it was informed that, in the event of wishing to participate in the study, the Physical Education teacher was responsible for collecting completed parental informed permission papers and informing the research team of the day on which they could attend the courses in-person to complete the questionnaire with the children who at the time were in the fifth or sixth grade of primary school.

To ensure understanding of the items, before the start of the questionnaire, a member of the research team and the Physical Education teacher of the center read each item and made sure that the participants stated that they understood all the questions. To avoid technical errors, the questionnaires were administered from tablets owned by the research team and prepared for this purpose. All information was gathered in an anonymous manner, and the average response time was 5 min. Data were collected between March, April, and May 2021. The response rates for fifth and sixth grade students were 3.2% and 2.9%, respectively. In addition, the valid response rate was 100% thanks to the previous introduction of the questionnaire to the students by the research team and the Physical Education teacher.

### 2.4. Statistical Analysis

The Statistical Package for Social Sciences (SPSS) version 23.0 for MAC was used to analyze the data collected in the questionnaires. The Kolmogorov Smirnov test was done to see if the data were normal. This test indicated that the assumption was not met, so nonparametric tests were used. Descriptive data are presented as median (Me) and interquartile range (IQR).

Variations in questionnaire items depending on gender and center location were investigated using the Mann–Whitney U test (Table 2). The Mann–Whitney U test was also used to look at differences in the median total score of the questionnaire as a function of gender and center location (Table 3).

The link between the questionnaire’s median score and the age variable was investigated using Spearman’s Rho test (Table 4).

The instrument’s reliability was evaluated using Cronbach’s Alpha. According to Nunnally and Bernstein [35], reliability levels of 0.60 to 0.70 are acceptable, whereas values of 0.70 to 0.90 are satisfactory.

## 3. Results

Table 2 displays descriptive statistics for each of the PACES questionnaire questions based on the median (Me) and interquartile range (IQR) for each sex and center location. To examine differences, the Mann–Whitney U test was utilized.

Table 3 shows the total sum and the median Likert score achieved in the PACES as a function of sex and center location.

Males scored higher than females, and the differences were statistically significant (*p* < 0.001). Students from rural schools outperformed those from urban schools when it came to the center’s location. There were also statistically significant differences (*p* = 0.021).

Using Spearman’s test, Table 4 reveals the relationships between PACES and age.

Finally, according to Nunnally and Bernstein (1994), the questionnaire’s reliability score was outstanding (Total = 0.90; Positive = 0.86; and Negative = 0.834).

## 4. Discussion

This research was carried out to determine the degree of satisfaction of fifth and sixth grade students when they engage in physical activity, also differentiating according to gender or location of the school. That students are satisfied with the physical activity they practice should be a premise that teachers should try to seek in their classes to achieve an optimal motivational and performance context. To find out whether this is the case or not, a questionnaire was passed around which collected different statements on whether there is a positive or negative satisfaction with respect to the students’ feelings. It can be said that there are many benefits to children’s satisfaction and enjoyment of physical activity that can lead to improvements in their level of moderate or vigorous physical activity [36], and improve their general health status, especially in the case of children with disabilities [37], or serve as a predictor of initiating or maintaining an active lifestyle in the future.

As for the items that refer to positive perceptions of physical activity, generally, the results are very good. For this reason, most students would choose PE as an elective, rejecting the possibility of reducing the number of hours of the subject [38]. In general, such high scores can be achieved from different strategies: Moreno-Murcia and Vera consider that there is a predictive relationship between intrinsic motivation and enjoyment in PE classes and the ability to predict improvements in satisfaction [39]; Invernizzi et al. state that a methodology based on guided discovery, problem solving through collaborative learning strategies, and direct application of instruction and task demand emphasize positive engagement in physical activity, increasing satisfaction levels with PE classes [40]. However, there are significant differences by sex in all the items that refer to positive feelings, with no differences according to the location of the school. In this sense, Lemes and colleagues found differences in the levels of physical activity, its intensity, and sports practice in school students between genders [41]. Moreover, Moore and Fry [42] pointed out that exercise ownership is the only mediator of satisfaction in the PE class, showing differences between sexes, which could explain the results of this study. Considering the diverse items referring to feelings of rejection towards physical activity, it could be said that they are satisfied with the physical activity they perform and that this does not vary independently of the location of the center or their sex. These results are similar to those of other authors who have also concluded in their studies that the way in which PE classes are taught is liked by most students, so they attach great importance to the subject and show high levels of satisfaction [38,43].

Conversely, significant differences were found in the sex variable in the positive and negative dimensions, as well as in the total score of the questionnaire. Previous research has already indicated that there may be differences in satisfaction in Physical Education classes depending on gender [44,45], this may be influenced by the heterogeneity of the groups, the size of the class, or the different physical activity needs of the students depending on their age [46,47]. If we focus on the differences obtained depending on the location of the school, McCaughtry and colleagues [48] noted a greater preparation of PE professors to develop their teaching activity in urban environments. Nevertheless, the satisfaction results of this study favor rural environments, which may be clearly influenced by the presence of physical environments more oriented to physical activity in comparison with cities [49].

Finally, the correlation between age and the PACES questionnaire is not significant; therefore, there is no relationship extracted between the age of the students surveyed and their scores on the different items of the questionnaire, although previous research has already suggested that these results vary according to the age of the students [41,44].

This research has had a balanced sample for the two dimensions that have been analyzed (sex and location of the center) but it could be a limitation not to have a dimension that analyzes the results of the questionnaire by course, something that would be very interesting to know how satisfaction varies throughout the school career. This is proposed as a future line of research.

## 5. Conclusions

The satisfaction of children with physical activity could be an indicator of their present or even future health since this will mark the adherence to healthy lifestyle habits. It is for this reason that the importance of this study, which shows, in general terms, that most students are satisfied with the physical activity they do, is even more important. 

Teachers should continue to focus on methodologies that motivate students intrinsically and, above all, emphasize that girls also feel included and motivated to do it, especially in the upper grades or in secondary education, where they tend to disengage. 

Since, as we have seen, there is no relationship between the age of the respondents and the score obtained in the different items, it will be necessary to continue reinforcing the tasks in the classroom and to demonstrate that physical activity is a faultless means of achieving academic performance in students.

## Figures and Tables

**Table 1:** The sample’s frequency distribution (N = 545).

Variable	Categories	N/M	%/dt
Gender	Male	277	50.8
	Female	268	49.2
Age	10	153	28.3
	11	245	44.7
	12	127	23.3
	13	20	3.7
Course	Fifth course	282	51.7
	Sixth course	263	48.3
Province	Cáceres	468	85.9
	Badajoz	77	14.1
Center surroundings	Rural	303	55.6
	Urban	242	44.4

M: Mean; dt: Typical deviation.

**Table 2:** Descriptive analysis and differences by sex and center location of the questionnaire items.

		Gender				Center Environment			
Item	Total	Female	Male			Rural	Urban		
When I’m Active…	M_e_ (IQR)	M_e_ (IQR)	M_e_ (IQR)	*p*	*r*	M_e_ (IQR)	M_e_ (IQR)	*p*	*r*
1. I enjoy it	5 (0)	5 (0)	5 (1)	<0.001 **	0.20	5 (0)	5 (0)	0.023 *	0.09
2. I get bored	5 (1)	5 (0.5)	5 (1)	<0.01 **	0.17	5 (1)	5 (1)	0.584	0.02
3. I don’t like it	5 (0)	5 (0)	5 (1)	0.010 *	0.11	5 (0)	5 (1)	0.219	0.05
4. I find it enjoyable	5 (1)	5 (1)	5 (1)	<0.01 **	0.21	5 (1)	5 (1)	0.194	0.05
5. It’s no fun at all	5 (0)	5 (0)	5 (0)	0.086	0.07	5 (0)	5 (1)	0.037 *	0.08
6. It gives me energy	5 (1)	5 (1)	5 (1)	<0.01 **	0.13	5 (1)	5 (1)	0.102	0.06
7. It depresses me	5 (0)	5 (0)	5 (0)	0.073	0.07	5 (0)	5 (0)	0.032 *	0.09
8. It is very pleasant	5 (1)	5 (0.5)	5 (1)	<0.01 **	0.22	5 (1)	5 (1)	0.240	0.05
9. My body feels good	5 (1)	4 (1)	5 (1)	<0.01 **	0.21	5 (1)	5 (1)	0.046 *	0.08
10. I get something extra	4 (2)	5 (1)	4 (2)	<0.01 **	0.29	4 (2)	4 (2)	0.037 *	0.08
11. It is very exciting	4 (2)	4 (1.5)	4 (2)	<0.01 **	0.20	4 (2)	4 (2)	0.033 *	0.09
12. It frustrates me	5 (1)	5 (1)	5 (1)	0.189	0.05	5 (1)	5 (1)	0.493	0.02
13. It’s not interesting at all	5 (0)	5 (0)	5 (1)	0.233	0.05	5 (0)	5 (1)	0.145	0.06
14. It gives me strong feelings	4 (2)	4 (2)	3 (2)	<0.01 **	0.23	4 (2)	3 (2)	0.036 *	0.08
15. I feel good	5 (1)	5 (0)	5 (1)	<0.01 **	0.17	5 (0)	5 (1)	0.001 *	0.14
16. I think I should be doing something else	5 (1)	5 (1)	5 (1)	0.083 *	0.07	5 (1)	5 (1)	0.001 *	0.14

Note: Me = median value; IQR = interquartile range; *r* = Pearson r coefficient. Note: Differences are significant at ** *p* < 0.01; * *p* < 0.05. Each score obtained in the dimensions is based on a Likert scale (1–5).

**Table 3:** Each dimension of the questionnaire was subjected to descriptive analysis.

		Gender				Center Environment			
	Me (IQR)	Male	Female	*p*	*r*	Rural	Urban	*p*	*r*
**PACES** **Positive**	4.4 (0.9)	4.5 (0.8)	4.2 (0.9)	<0.001 **	0.30	4.4 (0.9)	4.3 (0.8)	0.008 *	0.11
	**MeΣ** **(IQR)**	**Male**	**Female**	***p***		**Rural**	**Urban**	***p***	
**PACES** **Positive**	40 (8)	41 (7)	38 (8)	<0.001 **	0.30	40 (8)	39 (7)	0.008 *	0.11
	**Me (IQR)**	**Male**	**Female**	***p***		**Rural**	**Urban**	***p***	
**PACES** **Negative**	4.8 (0.6)	4.9 (0.6)	4.7 (0.7)	0.017 *	0.10	4.9 (0.6)	4.9 (0.7)	0.125	0.06
	**MeΣ** **(IQR)**	**Male**	**Female**	***p***		**Rural**	**Urban**	***p***	
**PACES** **Negative**	34 (4)	34 (4)	33 (5)	0.017 *	0.10	34 (4)	34 (5)	0.125	0.06
	**Me (IQR)**	**Male**	**Female**	***p***		**Rural**	**Urban**	***p***	
**PACES** **Total**	4.50 (0)	4.62 (0.56)	4.5 (1)	<0.001 **	0.24	4.56 (0.63)	4.46 (0.63)	0.021 *	0.09
	**MeΣ** **(IQR)**	**Male**	**Female**	***p***		**Rural**	**Urban**	***p***	
**PACES** **Total**	72 (11)	74 (9)	70.50 (11.75)	<0.001 **	0.24	73 (10)	71.50 (10)	0.021 *	0.09

Note: Me = median value; IQR = interquartile range; *r* = Pearson r coefficient. Note: ** *p* < 0.001; * *p* < 0.05 indicate that the difference is significant. Each dimension’s score is calculated using a Likert scale (1–5).

**Table 4:** Correlations between the dimensions and the age variable.

Dimensions	Age ρ (*p*)
PACES Positive	−0.06 (0.16)
PACES Negative	−0.08 (0.07)
PACES Total	−0.09 (0.03 *)

Note: *r* = Pearson r coefficient. Note: Correlation is significant at * *p* < 0.05. Each score obtained in the dimensions is based on a Likert scale (1–5).

## Data Availability

The datasets used during the current study are available from the corresponding author on reasonable request.
